# Cancer mortality in Brazil: Temporal Trends and Predictions for the Year 2030

**DOI:** 10.1097/MD.0000000000000746

**Published:** 2015-04-24

**Authors:** Isabelle R. Barbosa, Dyego L.B. de Souza, María M. Bernal, Íris do C.C. Costa

**Affiliations:** From the Graduate Program in Collective Health (IRB); Department of Collective Health (DLBDS); Department of Odontology, Federal University of Rio Grande do Norte, Natal, Brazil (IDCCC); Department of Microbiology, Preventive Medicine and Public Health, University of Zaragoza, Zaragoza, Spain (MMB).

## Abstract

Cancer is currently in the spotlight due to their heavy responsibility as main cause of death in both developed and developing countries. Analysis of the epidemiological situation is required as a support tool for the planning of public health measures for the most vulnerable groups. We analyzed cancer mortality trends in Brazil and geographic regions in the period 1996 to 2010 and calculate mortality predictions for the period 2011 to 2030.

This is an epidemiological, demographic-based study that utilized information from the Mortality Information System on all deaths due to cancer in Brazil. Mortality trends were analyzed by the Joinpoint regression, and Nordpred was utilized for the calculation of predictions.

Stability was verified for the female (annual percentage change [APC] = 0.4%) and male (APC = 0.5%) sexes. The North and Northeast regions present significant increasing trends for mortality in both sexes. Until 2030, female mortality trends will not present considerable variations, but there will be a decrease in mortality trends for the male sex. There will be increases in mortality rates until 2030 for the North and Northeast regions, whereas reductions will be verified for the remaining geographic regions. This variation will be explained by the demographic structure of regions until 2030.

There are pronounced regional and sex differences in cancer mortality in Brazil, and these discrepancies will continue to increase until the year 2030, when the Northeast region will present the highest cancer mortality rates in Brazil.

## INTRODUCTION

Despite the reduction in cancer mortality observed in developed countries in the last decades, the number of cases and deaths by cancer projected for the next 20 to 40 years will double the current numbers. Although the highest mortality rates are observed in developed countries, poor and developing countries already concentrate 80% of the burden of nontransmissible diseases in the world, and cancer will be the main cause of morbidity and mortality within the next decades in these regions.^1^

According to data and estimates of the World Health Organization, the number of deaths related to cancer increased from 6 million in the year 2000 to 7.6 million in 2007, revealing an increment of 32% in the magnitude of deaths between 2000 and 2007. In 2007, cancer was responsible for 13% of all deaths around the world.^2^ Projections for the year 2030 indicate the occurrence of 26 million new cases and 17 million deaths by cancer. These projected numbers are widely explained by demographic growth and the aging process of populations, especially in least developed regions of the planet.^3^

In Brazil, starting in the 1960s, several factors produced a fast epidemiological transition, increasingly exposing the population to the risk of chronic diseases. These factors were increase in income, industrialization and mechanization of production, urbanization, new lifestyles and reproductive patterns, better access to food in general, including processed food, and the globalization of unhealthy habits.^4,5^ In parallel, the patterns of distribution of risk factors were deeply affected by demographic changes in Brazil: the number of elderly changed from 3 million in 1960 to 7 million in 1975, and 20 million in 2008—an increase of almost 700% in <50 years. Consequently, age-related diseases started to gain importance for society as a whole.^6^

As a result of this process, in Brazil, cancer is the second most common cause of death, after cardiac and cerebrovascular diseases. In 2014, there were approximately 395,000 new cancer cases, 205,000 in men and 190,000 in women. The most incident types of cancer are prostate, lung, colon, and rectum. In women, the most common cancers are breast, colon and rectum, cervix, lung, and thyroid.^7^

The incidence patterns of Latin American and Caribbean countries are directly related to social and economic inequities, whereas mortality reflects the structure and organization of health systems in each country.^8^ The increase in cancer incidence in addition to disproportionate mortality rates is directly related to the organization of screening services, prophylactic actions of immunization and control of risk factors, and availability and access to diagnostic and treatment methods for cancer. Control of risk factors along with organization of health services has become, therefore, a challenge regarding cancer control in Latin America.^9^

Keeping in mind the relevance of the burden of cancer in the Brazilian population and its potential for health system actions, monitoring of mortality trends throughout time and knowledge on future mortality patterns are relevant for the planning and evaluation of cancer control politics as well as the implementation of methods for early detection and treatment, directed to the most vulnerable areas.

This study had the objective of analyzing the temporal trends of cancer mortality in Brazil in the period 1996 to 2010 and calculated the projection of mortality in the period 2011 to 2030, verifying the occurrence of regional and sex differences.

## METHODS

An epidemiological study was carried out, of temporal-series retrospection, based on secondary data collected from the Mortality Information System (MIS) of the Department of Informatics of the Unified Health System (Datasus). Deaths from cancer in Brazil were analyzed, registered in the period 1996 to 2010, according to sex (All cancers deaths excluding non-melanoma skin cancer).

The temporal trends for cancer mortality in Brazil and geographic regions were analyzed, and mortality projections until the year 2030 were calculated, in 5-year groups, for the periods 2011 to 2015, 2016 to 2020, 2021 to 2025, and 2026 to 2030.

The *Joinpoint* regression analysis was utilized to analyze mortality trends, through the software Joinpoint Regression Program (National Cancer Institute, Bethesda, MD), version 4.1.0., of April 2014. The objective of the analysis was to identify the occurrence of possible joinpoints where a significant change in trend occurred.

The applied method identified joinpoints based on the model with a maximum of 3 change points. The final model selected was the most adjusted model, with annual percentage change (APC) based on the trend of each segment, estimating whether these values were statistically significant to a 0.5 level. The significance tests utilized were based on the Monte Carlo permutation method and on the calculation of the APC of the ratio, utilizing the logarithm of the ratio.

The average annual percent change (AAPC) was calculated to quantify the cohort trend in the analyzed years. AAPC is based on the geometric accumulated mean of the APC trends, with equal weights for the lengths of each segment during the fixed interval.

When describing trends, the terms “significant increase” or “significant decrease” mean that the slope of the trend is statistically significant (*P* < 0.05). For nonsignificant trends, the term “stable” was used.

Predictions were made for each period utilizing the age-period-cohort model of the Nordpred program (Cancer Registry of Norway, Oslo, Norway), within the statistical program R. Data were compiled in 5-year blocks and the limit age group considered for analysis was the first with >10 cases for the combined period.

The results of the predictions are presented for the total of observed and expected deaths, per period in Brazil and its 5 geographic regions. For each period, adjusted mortality rates were calculated on the basis of the standard world population for global comparisons, expressed per 100,000 inhabitants per year (ASW/100,000 inhab).^10^ The adjusted mortality rates were also calculated for each age group considered in the study.

The annual changes in the number of cases in the last projected period (2026–2030) in comparison to the last observed period (2006–2010) were calculated, wherein the proportion of change occurred in terms of alterations in risks or demographics (size or structure of population). These 2 components can be different from 0 and present a positive or negative direction. The calculation can be expressed as follows^11^: Δ_tot_ = Δ_risk_ + Δ_pop_ = (N_fff_ − N_off_) + (N_off_ − N_ooo_)

where *Δ* tot is the total change, *Δ* risk is the change in function of risk, *Δ* pop is the change in function of the population, Nooo is the number of observed cases, Nfff is the number of projected cases, and Noff is the number of expected cases when mortality rates increase during the observed period.

Data for female and male populations in Brazil and geographic regions, according to age groups, were obtained from census information (2000 and 2010), counting (1996), and inter-census projections of the Brazilian Institute of Geography and Statistics.

In the analysis of sex ratio, the quotient of the standardized mortality rates for men and women was calculated for the observed (2006–2010) and projected (2026–2030) periods.

This research used secondary data available on official websites of the Ministry of Health of Brazil, without identification of subjects, not requiring approval by the ethics committee, in accordance with Resolution 466/2012 of the National Health Council in Brazil.

## RESULTS

**FIGURE 1:**
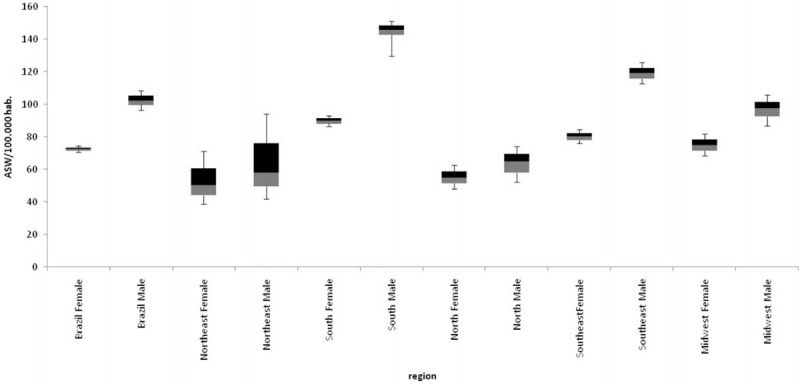
Standardized cancer mortality rates for Brazil and its geographic regions, according to sex, for the period 1996–2010.

Between 1996 and 2010, 2,023,038 deaths occurred due to cancer in Brazil, with 53.9% of the deaths affecting the male sex and 46.1% affecting the female sex. The standardized mortality rates for men in Brazil varied from 96.32 deaths/100,000 inhabitants for the year 1996, to 99.68 deaths/100,000 inhabitants in 2010. For women, these rates varied from 70.82 deaths/100,000 inhabitants in 1996, to 74.48 deaths/100,000 inhabitants in 2010. The mortality rates for the male sex in the South and Southeast regions were above the registered rates for Brazil in the period (Figure 1).

Joinpoint analysis verified stability for female mortality (APC = 0.4%); however, there was a significant increasing trend for the male sex, with APC = 1.2% between 1996 and 2008, with a consequent reduction, although nonsignificant. The Southeast region registered a significant decrease in female (APC = −0.7%) and male (APC = −0.8%) mortalities. The Northeast region presented significant increasing mortality trends for both sexes starting from 2003 (Table 1).

**TABLE 1:**
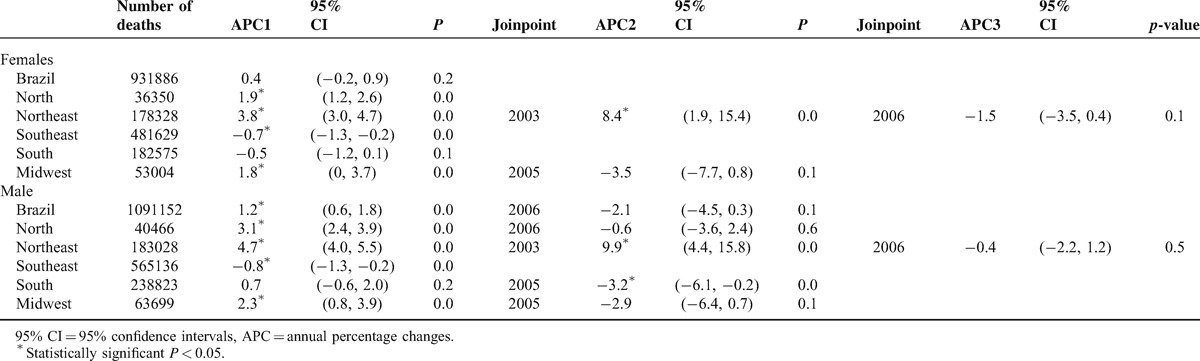
Temporal Trends for Cancer Mortality in Brazil and Regions: Number of Deaths, APC, Confidence Interval, and Joinpoint Year

In the cohort analysis for 15 years, the North and Northeast regions presented increasing trends, the Southeast region presented decreasing trends, and the Midwest and Southeast regions presented stable rates (Table 2).

**TABLE 2:**
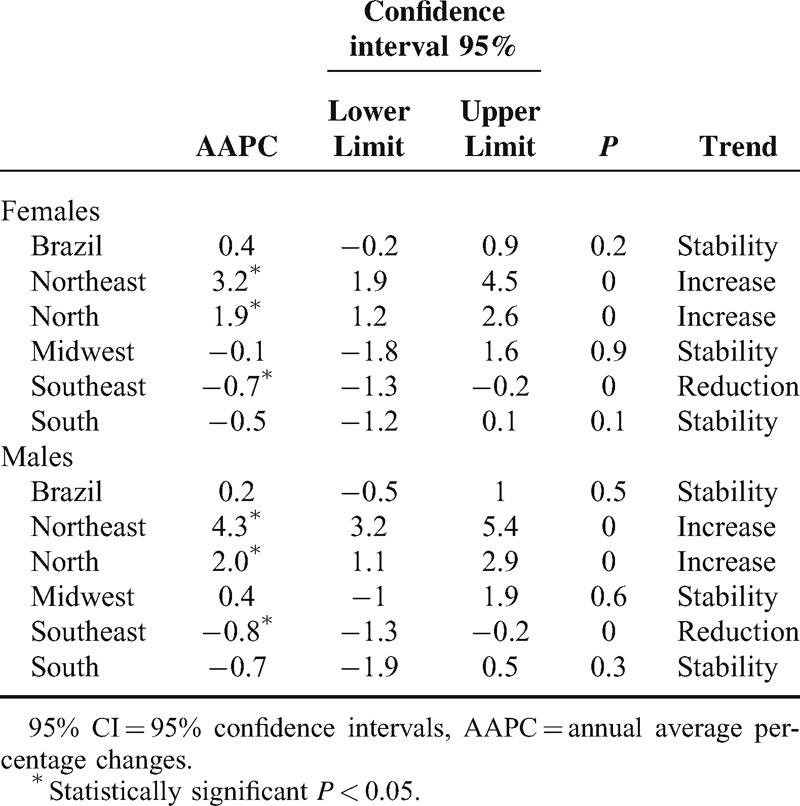
AAPC of Cancer Mortality Rates for Men and Women in the 15-year Cohort (1996–2010) for Brazil and Regions

Table 3 presents the number of deaths and standardized mortality rates for the observed and projected periods. Analysis of Brazilian data for the 5-year period, 2026 to 2030, resulted in the projection of 690,394 female deaths by cancer. For the male sex, 757,806 deaths are expected between 2026 and 2030. No considerable variations are expected for female mortality rates; however, for the male sex, a reduction in mortality rates will be observed, which will vary between 99.02 deaths/100,000 inhabitants in the last period of 2006 to 2010 and 88.04 deaths/100,000 inhabitants in the period 2026 to 2030. For the North and Northeast regions, there will be an increment in mortality rates until the year 2030 for both sexes, whereas for the South, Southeast, and Midwest regions, there will be a reduction in mortality rates throughout the projected period.

**TABLE 3:**
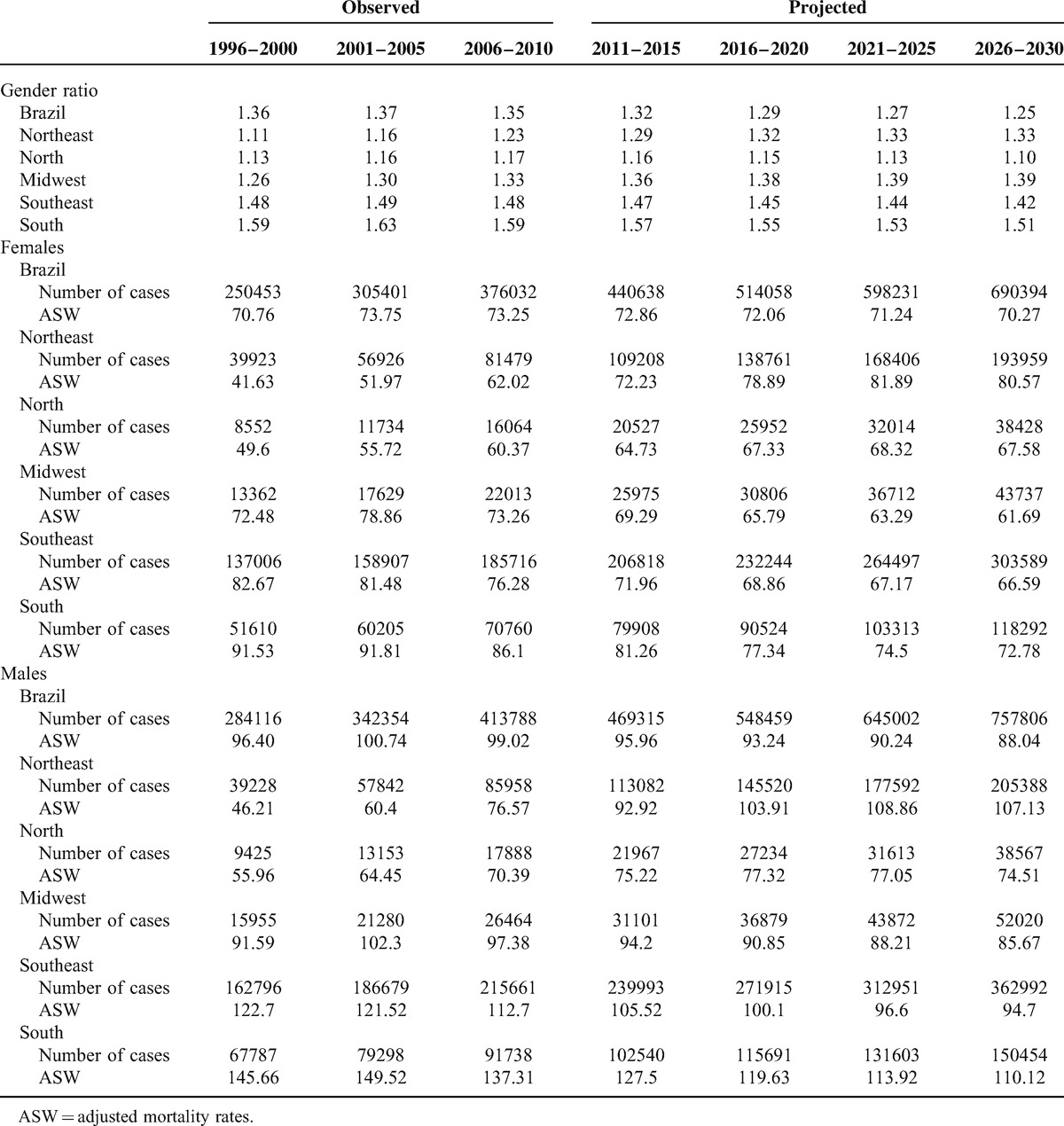
Cancer Mortality in Brazil and Regions: Gender Ratio, Number of Observed and Projected Deaths, and Mortality Rates Adjusted to the World Population (ASW /100,000 inhab)

These data can be reinforced by the results of the mortality ratio between sexes when the last observed period is compared with the last predicted period: Brazil (from 1.35 to 1.25); Northeast (from 1.23 to 1.33); North (from 1.17 to 1.10); Midwest (from 1.33 to 1.39); Southeast (from 1.48 to 1.42); South (from 1.59 to 1.51). These results show increasing mortality trends for the male sex in the Northeast and Midwest regions, whereas for Brazil and remaining geographic regions, the scenario will be of increasing mortality for the female sex.

Table 4 shows the projected deaths according to the influence of risks and population structure in Brazil and its geographic regions in the year 2030. This calculation shows that in 2030, there will be a higher number of deaths than expected, if population structure and risks maintain the 2010 levels. In majority, the projected numbers are explained by the variation in demographic structure, with an exception for the Northeast region, where changes in risk will mostly explain the mortality rates in 2030 for both sexes.

**TABLE 4:**
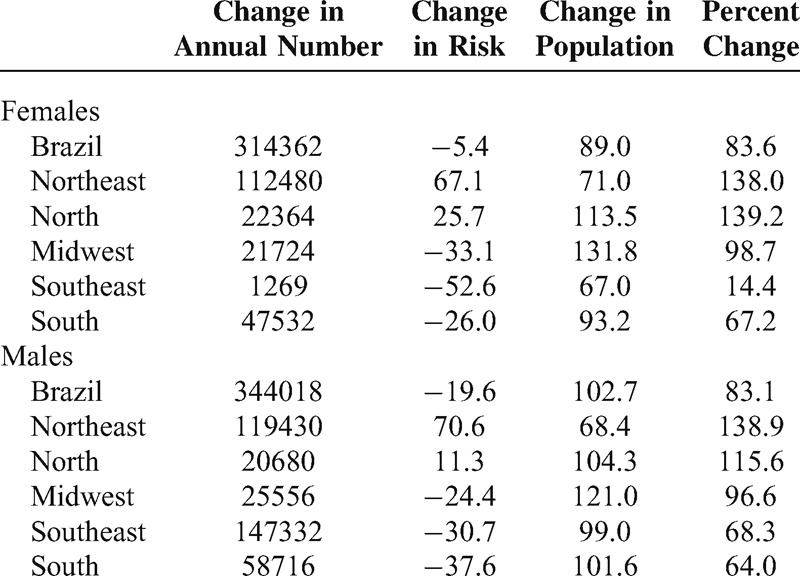
Change in the Number of Deaths Between 1996–2010 and 2026–2030, and Changes Relative to Structure of Population and Risk

## DISCUSSION

The results obtained reveal great differences in cancer mortality patterns in the Brazilian regions, highlighting the significant increasing trend throughout the historical series and increasing projected mortality trends until the year 2030 for the North and Northeast regions of the country. Stable and/or decreasing trends were verified for the South, Southeast, and Midwest regions. Differences in sex mortality also deserve mention, with reduction in male mortality until the year 2030.

In the cancer mortality estimates for 40 European countries in 2012, the standardized mortality rate for men was 222.6 deaths/100,000 inhabitants, and for women, 128.8.^12^ For the same year, in a population-based research carried out in India, a mortality rate of 97.7 deaths/100,000 inhabitants was registered for men, and 95.5 deaths/100,000 inhabitants for women.^13^ Comparison of these results indicates that cancer mortality rates in Brazil are well below the rates for developed countries, and in a similar range of the rates verified for developing countries.

This general trend of reduction in standardized cancer mortality rates in Brazil is corroborated by another Brazilian study that observed similar behavior in the state capitals, indicating reduction in age-specific rates until the age of 69 years, for both sexes.^14^ However, the stable and/or decreasing trends for cancer mortality in Brazil observed herein are in the opposite direction of the perspectives for Latin America, following the trends of more developed countries. In Latin America, estimates indicate an incidence increase of 72% and an increase of 78% in the mortality of men between 2012 and 2030. For women, these rates are, respectively, 62% and 74%.^8^ According to the national cancer statistics of Korea, the mortality for all combined cancers showed a decrease of 2.7% per year in the period 2002 to 2010.^15^ Cancer mortality analysis in Canada between 1970 and 2007 revealed a decline in mortality trends for all cancers combined (AAPC = −0.3% 95% confidence interval [CI] −0.4, −0.3).^16^ In Japan, mortality trends are decreasing since the mid-1990s (APC = −1.3% 95% CI −1.4, −1.3).^17^

The decreasing mortality trends across cancers, observed herein, are the result of the sum of the behaviors of individual cancer trends. Among the epidemiological changes suffered by Brazil in recent decades, a specific behavior in the mortality profile for some types of cancer must be highlighted. Examples include prostate, lung, and colorectal cancers, which in the last decades remain with ascending trends.^18–21^

Analysis of the GLOBOCAN data for 53 countries verified reductions in prostate cancer mortality rates in North America, Oceania, West Europe, and part of North Europe, whereas increases have been observed in Central and East Europe, South America, and part of Asia and Africa.^1^ In a study carried out in Brazil, which analyzed deaths by prostate cancer occurred between 1980 and 2010, increasing temporal trends were verified in mortality rates across all geographic regions of the country, with an average increase of 2.8% per year.^1^

Mortality behavior is influenced by at least 2 variables: incidence and survival. It is conceivable that the incidence of a specific type of cancer increases throughout time, but due to significant advances in secondary prevention and treatment, survival also increases to the point of translating into a reduction in mortality.^14^ In the equation incidence-mortality-survival, prevalence and distribution of risk factors in the population—as well as organization and accessibility to the health system—influence current and future epidemiological patterns for cancer.^8^

Some factors associated with the increase of cancer incidence and mortality are related to the development of cancer, and close vigilance of these population characteristics will enable the elaboration of prevention and control strategies. This is the case of the consumption of tobacco, alcoholism, reproductive and sexual characteristics, prevalence of cancer-related infections, hormone therapy, diet habits, physical activity, and behaviors related to occidental or high-income lifestyles.^1,12^

Therefore, preventive measures must be implemented to reduce the burden of cancer, in addition to strategies for the control of the consumption of tobacco (related to lung cancer, among other types of cancer), promotion of health eating habits (preventing stomach and intestine cancers, among others), and vaccination against the human papillomavirus and hepatitis (against cervical and liver cancers). Equally, the adoption of healthier lifestyles, which include adequate diet and physical exercise, will allow for a better control of breast, prostate, and intestine cancers. These measures present growing importance especially in countries such as Brazil, currently in an economic transition process that progressively drags into the country the global burden of cancer, as observed in economically developed countries.^7^

Other factors that can influence cancer mortality rates among different population groups are socioeconomic differences manifested in different aspects of the epidemiological profile. Evidence demonstrate that for lower socioeconomic level groups, there is elevated cancer mortality in general due to a greater proportion of late diagnosis of cancer that could be detected in earlier stages through screening. Also, there is a higher degree of difficulty in accessing adequate diagnosis and treatment, worse prognosis, and lower survival after diagnosis of cancer, higher death risk by cancer in general, and by potentially curable cancer types.^22–24^

Another critical point in Brazil is the organization of the health system for care of cancer patients. Latin American countries present fragmented health systems that operate with minimal structure to provide care, which, in general, is translated into emergency support. The global crisis affects the planning and the destination of resources to health systems, limiting development. The unequal allocation of resources, concentration of healthcare professionals in more developed urban centers, and low investments in equipment and infrastructure corroborate for the reproduction of socioeconomic inequities in the care of cancer patients.^8^

This situation is reflected in the unequal distribution of the hierarchic levels of care for cancer patients in Brazil. There are disparities between the areas with better urban structures (South and Southeast regions) that count with well-equipped health systems orderly distributed in the territory, and areas with absence of intermediary hierarchic levels (North and Northeast regions).^25^

The increase in cancer mortality expected for the poorer regions can be possibly explained by late diagnosis and inadequate therapeutic institution, resulting in mortality rates similar or even superior to those of developed countries. Precariousness is related to screening (when applicable), the equipment for diagnosis, stage of disease at time of diagnosis, available treatment methods, and, as a consequence, impact on survival.^26^

## CONCLUSIONS

The results of this study show that Brazil registers lower cancer mortality rates than developed countries, similar to those of developing countries. There are pronounced regional and sex differences in cancer mortality in Brazil, and these discrepancies will continue to increase until the year 2030, when the Northeast region will present the highest cancer mortality rates in Brazil. One of the limitations of this study is the short period of temporal series to carry out the mortality trend analysis. Regarding the death registry in Brazil, it must be mentioned that there were issues in the past with the reliability of data especially for the North and Northeast regions; however, advances in the mortality information system have been implemented since 2000.
